# Probing real-world Central European population midfacial skeleton symmetry for maxillofacial surgery

**DOI:** 10.1007/s00784-023-05185-x

**Published:** 2023-08-03

**Authors:** Adrian Franke, Elisabeth Claudia Hofmann, Anna Steinberg, Günter Lauer, Hagen Kitzler, Henry Leonhardt

**Affiliations:** 1 Department of Oral and Maxillofacial Surgery, University Hospital Carl Gustav Carus, 01304 Dresden, Germany; 2 Department of Diagnostic and Interventional Neuroradiology, University Hospital Carl Gustav Carus, Dresden, Germany

**Keywords:** Symmetry, Patient-specific implant, Midface, Maxillofacial, Computer tomography

## Abstract

**Objectives:**

Symmetry is essential for computer-aided surgical (CAS) procedures in oral and maxillofacial surgery (OMFS). A critical step for successful CAS is mirroring the unaffected side to create a template for the virtual reconstruction of the injured anatomical structure. The aim was to identify specific anatomical landmarks of the midfacial skeleton, to evaluate the symmetry in a group of the real-world Central European population, and to use these landmarks to assess midfacial symmetry in CT scans.

**Material and methods:**

The retrospective cross-sectional study defined landmarks of the midface’s bony contour using viscerocranial CT data. The distances of the skeletal landmarks (e.g., the frontozygomatic suture and temporozygomatic suture) of the left and right sides from the midline were measured and statistically compared. Midfacial symmetry for reference points was defined as a difference within 0 mm and their mean difference plus one standard deviation.

**Results:**

We examined a total of 101 CT scans. 75% of our population shows symmetrical proportions of the midface. The means of the differences for the left and right sides ranged from 0.8 to 1.3 mm, averaging 1.1 ± 0.2 mm for all skeletal landmarks. The standard deviations ranged from 0.6 to 1.4 mm, with a computed mean of 0.9 ± 0.3 mm.

**Conclusion:**

We established a methodology to assess the symmetry of the bony midface. If the determined differences were equal to or lower than 2.5 mm in the mentioned midfacial skeletal landmarks, then the symmetry of the bony midface was considered present, and symmetry-based methods for CAS procedures are applicable.

**Clinical relevance:**

Many CAS procedures require facial symmetry. We provide an easy-to-apply method to probe for symmetry of the midface. The method may be used for population-based research, to check for proper reduction of fractures after reposition or to screen for symmetry prior to CAS planning.

## Introduction

### Background

Symmetry is essential to the face’s aesthetics and interpersonal interactions [1]. It also relates to computer-aided surgical (CAS) procedures in oral and maxillofacial surgery (OMFS) because they are based on symmetry. If no facial symmetry or extensive defects in the facial skeleton are present, other methods for creating patient-specific implants (PSIs) must be applied, such as the superimposition strategy, where a model of a standard skull may be utilised as a template to replace missing or significantly asymmetric parts, and PSI manufacturing can proceed regularly [2]. Alternatively, the initial situation of the facial skeleton can be printed three-dimensionally, and PSIs can be moulded by hand on the printed model [3].

CAS procedures require the support of computers for planning and performing operations and even computer-assisted design and manufacturing. An essential step for successful CAS planning is mirroring the healthy, unaffected side to create a template for the virtual reconstruction of the injured anatomical structure. This step only satisfactorily works if the facial skeleton is symmetrical. Examples of CAS procedures include planning navigation-assisted operations in facial trauma surgery or orbital reconstruction or designing and manufacturing PSIs. PSIs offer defect-oriented and highly individualised care that enables more precise and less invasive interventions [4]. Regarding facial skeleton reconstructions, a suggested workflow is clinical assessment followed by three-dimensional imaging for bony structures. The next step includes planning and simulation using special software to simulate the desired surgical outcome. This step also includes the virtual fitting of implants. Then, based on this planning and simulation, a patient-specific model, including virtual reconstructions, serves as a template for producing PSIs. In theatre, the created implant needs to be transferred as precisely as possible to the planned position. Ideally, position control is achieved by using intraoperative three-dimensional imaging to validate the result and provide adequate quality control [5].

There are plenty of suggestions to define symmetry. Bilateral symmetry is when the left and right sides are mirrored copies of each other, as is true for individuals of the animal kingdom [6]. Depending on the distribution of asymmetry in a population, terms like directional asymmetry, anti-asymmetry and fluctuating asymmetry are historically mentioned [7]. Another approach to describing symmetry can be to define it as static or dynamic. Static symmetry refers to an exact arrangement of several elements to one another mirrored along an imaginary midline. However, an object close to perfect symmetry appears “boring” or unnatural. In nature, there is no static symmetry. However, dynamic symmetry is present. Dynamic symmetry occurs when similar, but not identical, halves of a whole face each other along an imaginary line, for example, the left and the right side of the face. In nature, this kind of symmetry is ubiquitous, considered “lively” and “beautiful”, and appears more natural to the observer [8]. The treating surgeon should heed dynamic symmetry in patients when planning operations. Since facial aesthetics and harmony are of particular relevance to many people and the bony anatomy significantly influences the morphology of the soft tissue, it is advisable to plan treatment based on symmetry.

Numerous investigations deal with finding either a way to objectively evaluate the symmetry of the face or a universally valid method to construct the median line of symmetry of the face. Current studies mainly measure soft tissue symmetry by photogrammetry or the bony symmetry of individual facial regions [9–11].

### Objectives

Our study is aimed at identifying specific anatomical landmarks of the midfacial skeleton in CT scans, evaluating the midfacial symmetry in a group of the real-world Mid-European population, and using them to assess midfacial symmetry in any patient’s CT scan reliably. Furthermore, we establish a simple methodology to differentiate between bony midfacial symmetry and asymmetry in CAS planning.

## Material and methods

### Study design, setting, and participants

This retrospective cross-sectional study used viscerocranial CT data of patients without deformation or developmental anomaly scanned by the Institute of Diagnostic and Interventional Neuroradiology at the University Hospital Dresden from 2017 to 2019. Inclusion criteria were (1) absence of bony destruction or other relevant bone pathology of the skull and the midface and (2) adult patients only. Scans were excluded, for example, if midfacial injuries or asymmetry-producing pathologies like clefts, fibrous dysplasia, chronic sinusitis, or similar were evident.

All data were acquired using a SOMATOM Definition Edge Scanner (Siemens Healthineers, Erlangen, Germany): viscerocranial spiral computed tomography; 2 × 64 × 0.6 mm collimation; and multiplanar primary and secondary reconstructions in soft tissue and bone windows.

### Variables and data measurement

The extension of the examined midfacial region is defined as the entire viscerocranium, excluding the mandible and the tooth-bearing part of the maxilla. Experienced consultants of oral and maxillofacial surgery in our department defined skeletal landmarks that contribute significantly to the midface’s bony contour, which can be easily found in the bone window of CT datasets (Table 1). Measurements were undertaken by a single investigator on the experience level of a registrar.

**Table 1:** Skeletal landmarks to analyse midfacial symmetry

Anatomic point	Abbreviation	Description
Frontozygomatic suture	SF	Most lateral point of the suture between the zygomatic and frontal bone
Temporozygomatic suture	ST	Most lateral point of the suture between the zygomatic and temporal bone
Infraorbital canal	CI	Most lateral point of the bony canal of the orbital floor
Crista lacrimalis posterior	CL	Most lateral point of the bony crest of the lacrimal bone of the orbital floor
Lateral orbita	OL	Most lateral point of the outer border of the orbita
Medial orbita	OM	Most medial point of the inner border of the orbita

The CT data of the selected scans were analysed using the clinical, radiological diagnostic IMPAX EE software (v20190821_0813, Agfa HealthCare N.V.) for DICOM image presentation, reconstruction and postprocessing. For imaging, ultra-sharp (H70h) and sharp (H60s) convolution kernels, so-called “bone windows”, were used [12].

**Fig. 1:**
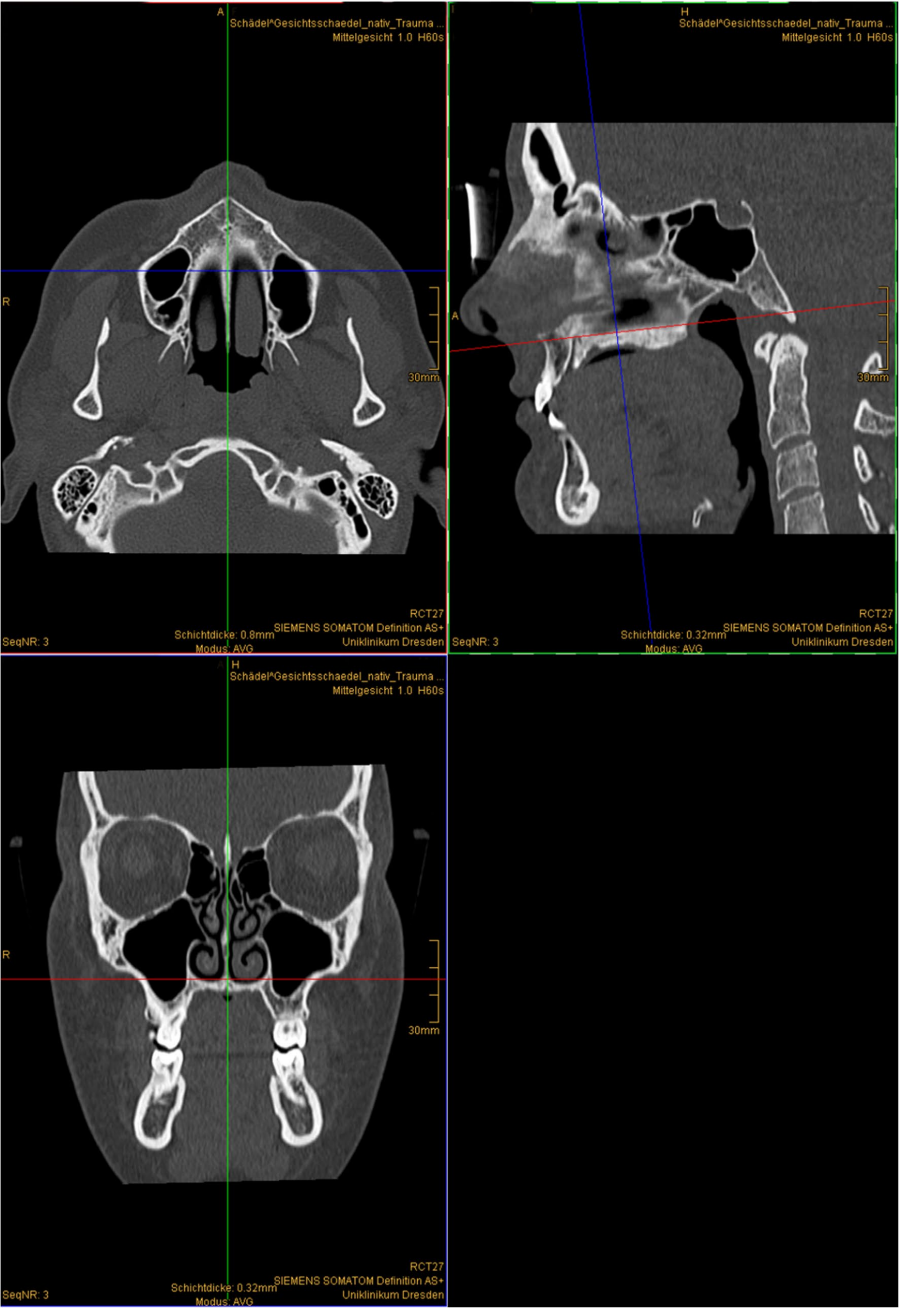
Example-planes for the definition of the median plane in CT-scan

We used the IMPAX EE “Extended Multi-planar Reconstruction Plugin” to receive simultaneous views of the coronary, axial and sagittal planes. The correct positioning for the measurements was assured by first defining the median plane according to our set anatomic reference points (Table 2) for the horizontal direction and adjusting the sagittal tilt to the maxillary plane (Fig. 1).

**Table 2:** Anatomic reference points to determine the median plane

Reference point	Abbreviation	Description
Anterior nasal spine	ANS	Most anterior point of the anterior nasal spine
Posterior nasal spine	PNS	Most posterior point of the posterior nasal spine
Sella turcica	S	Centre of the Sella turcica
Nasion	N	Most anterior and cranial point of the nasofrontal suture

In order to ensure that the median plane does not shift when scrolling through the slices, we synchronised the coronary, axial and sagittal planes with the “Synchronise” function.

**Fig. 2:**
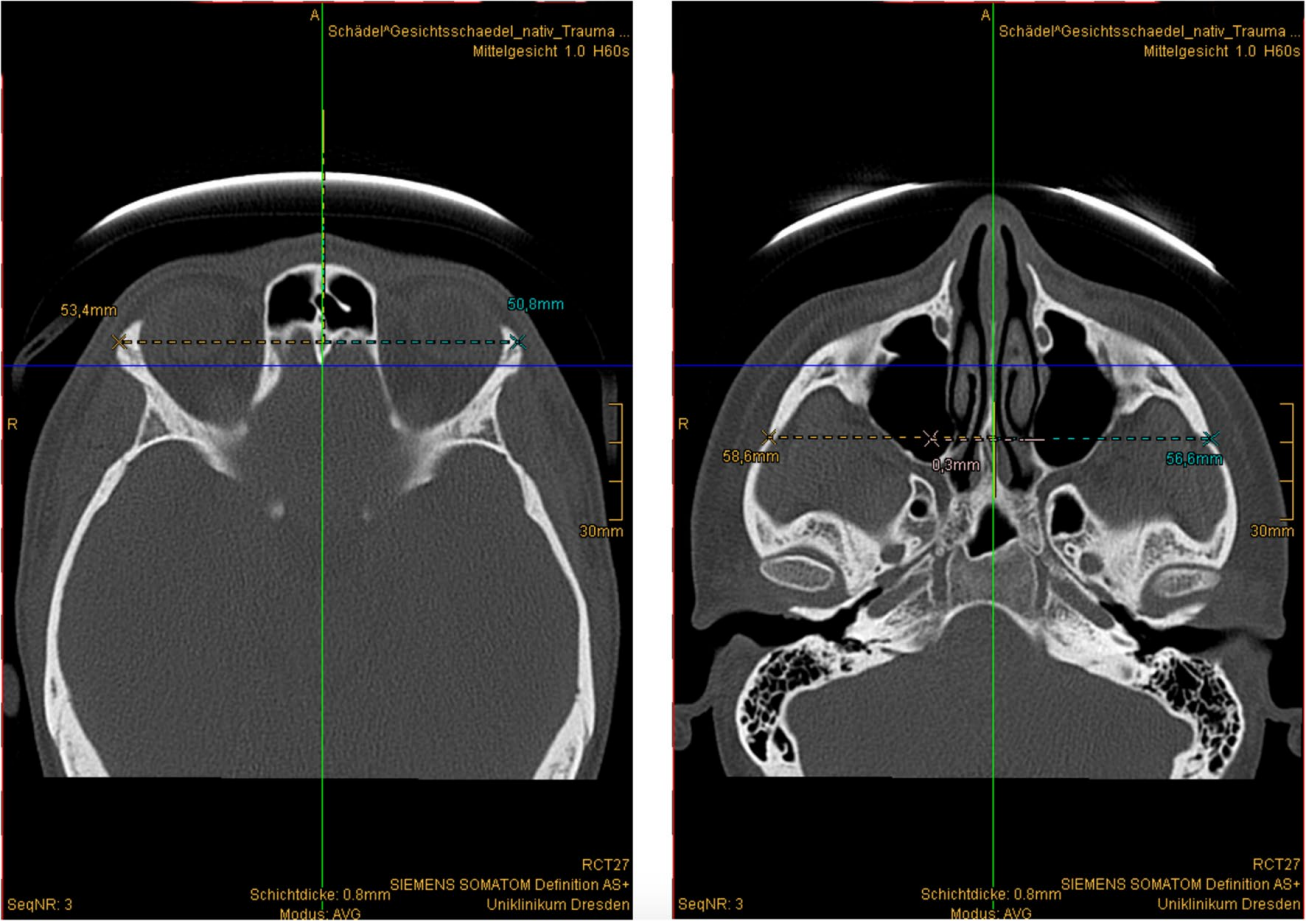
Distance measurement in the transverse direction. Left: frontozygomatic suture, right: temporozygomatic suture

**Fig. 3:**
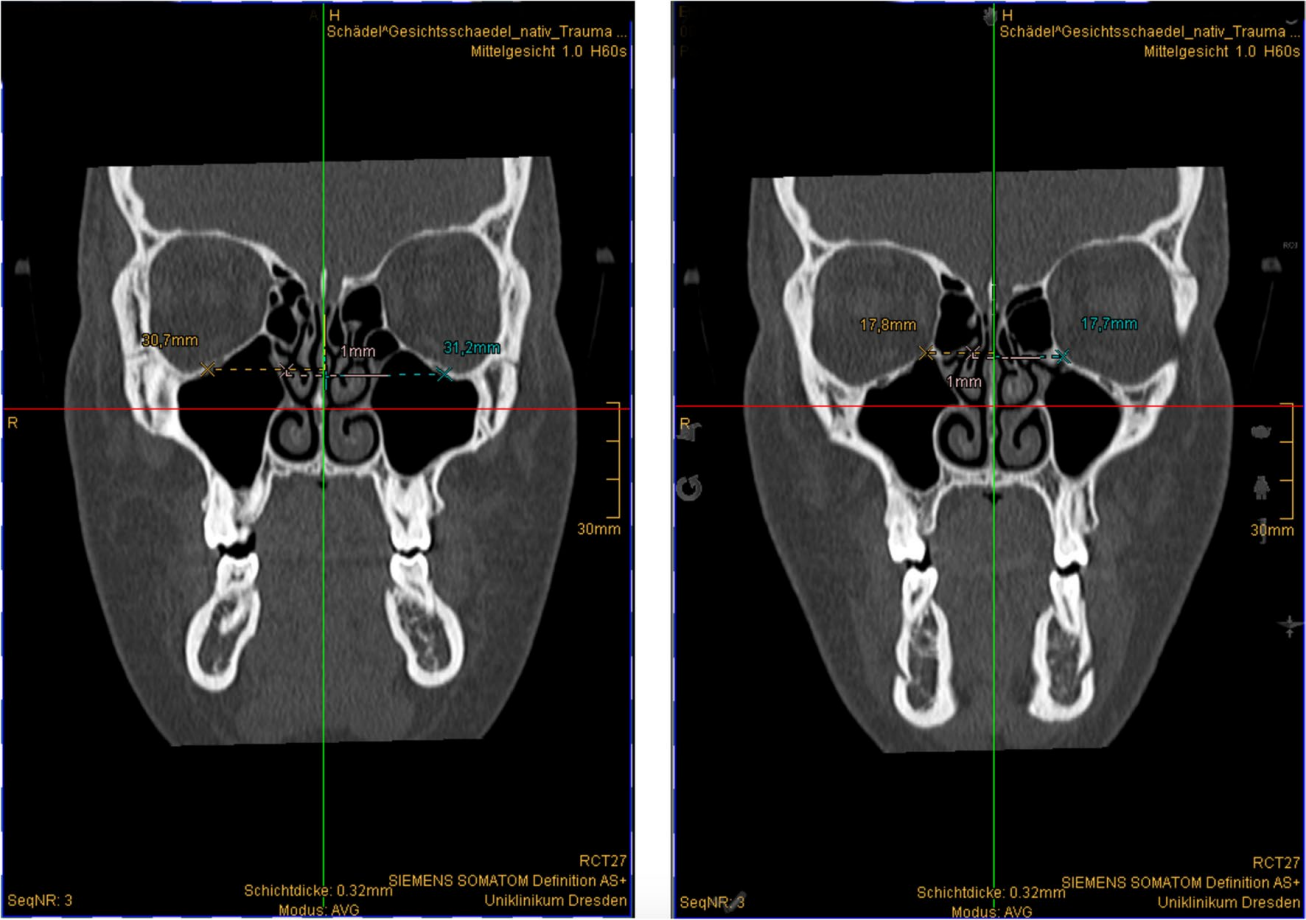
Distance measurement in the transverse direction. Left: infraorbital canal, right: crista lacrimalis posterior

**Fig. 4:**
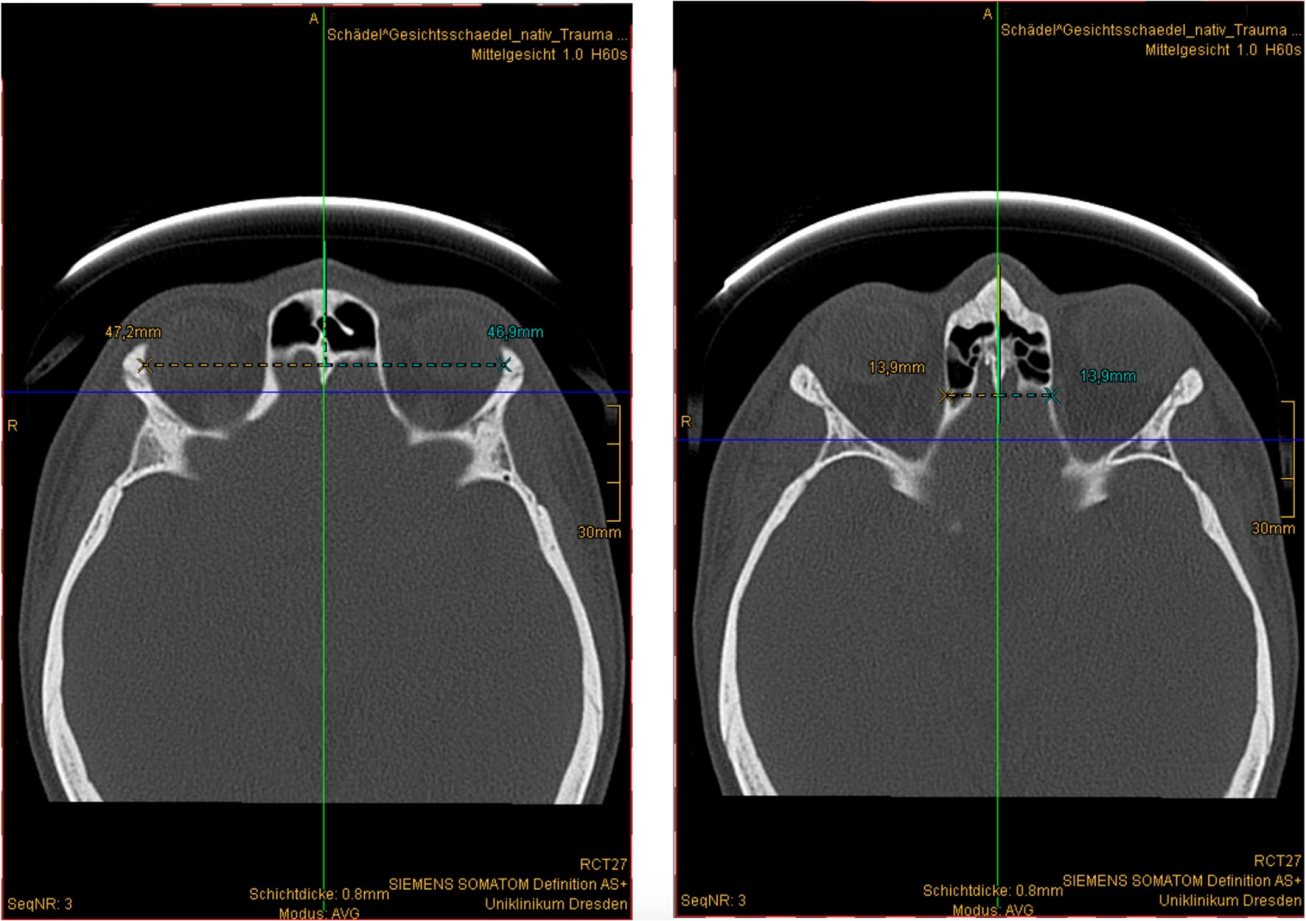
Distance measurement in the transverse direction. Left: lateral orbita, right: medial orbita

We measured the distances of the skeletal landmarks of the left and right sides from the midline in the axial or coronary plane with the “Measure normal distance in the image” function. We separately collected the measured lengths in millimetres from each CT scan (Figs. 2, 3, and 4).

### Bias and reproducibility

To confirm the reliability of our results, intra- and intraobserver bias was tested by randomly selecting patients from the study sample database and obtaining repeated measures (*n* = 10 for each skeletal landmark) for the differences of measures between the left and right sides at two different time points by two separate investigators (a registrar and a consultant).

### Statistical methods

Data were collected and statistically analysed using SigmaSTAT (Systat Software GmbH, Erkrath, Deutschland). For all the distances measured between the midline and the reference points, the minimum, maximum, mean, and standard deviation were calculated for the left and right half of the skull. Also, the mean differences between the left and right sides were computed. After proof of a normal curve of distribution, a paired two-sample *t*-test was applied to compare the opposing sides. A significance was assumed for *p* < 0.05. Minimum, maximum, means, and standard deviations for the differences between both sides were calculated and used as reference values to define symmetry.

We considered subjects to be symmetrical when all the reference points were symmetrical, or a maximum of one reference point was asymmetrical. The presence of symmetry for reference points was defined as a difference between the right and left sides in the transverse direction within 0 mm and the mean difference of the left and right sides plus one standard deviation. All other outcomes were considered asymmetrical subjects.

## Results

### Participants and descriptive data

A total of *n* = 101 CT scans were provided by the Institute of Diagnostic and Interventional Neuroradiology at the University Hospital Dresden and subsequently examined. There were 55 male (54.5%) and 46 female (45.5%) patients in the cohort. Overall, the age ranged from 19 to 93 years, with a mean age of 53.4 ± 19.4 (95% CI 49.6 to 57.2). The men in our study were between 19 and 93 years old, averaging 53.4 ± 17.2 (95% CI 48.8 to 58.1) years and the women were between 19 and 88 years old, averaging 53.3 ± 21.9 (95% CI 46.8 to 59.8) years. There was no statistical difference (*p* = 0.90) between the mean ages of men and women.

### Reproducibility

We measured all the reference points mentioned above for each CT scan according to the method described. Intra- and interobserver reliability and reproducibility were ascertained. After performing repeated measure one-way ANOVA including Tukey’s multiple comparison tests, there were no significant differences between the two examiners proving intra- and interobserver reliability. There was no statistical difference in the measured midfacial skeletal landmarks between the two time points (Table 3).

**Table 3:** Reproducibility for skeletal landmarks between two time points

Skeletal landmark	Time point 1		Time point 2		Difference between time points 1 and 2
	Mean ± SD (mm)	95% CI (mm)	Mean ± SD (mm)	95% CI (mm)	Mean of differences ± SD, 95% CI (mm), significance
Investigator 1					
SF	1.1 ± 0.8	0.5 to 1.6	0.8 ± 0.5	0.4 to 1.2	0.3 ± 0.5, − 0.1 to 0.6, *p* = 0.15
ST	1.0 ± 0.8	0.5 to 1.6	1.1 ± 0.9	0.5 to 1.7	− 0.1 ± 0.5, − 0.4 to 0.3, *p* = 0.76
CI	2.1 ± 1.2	1.3 to 3.0	1.9 ± 0.9	1.3 to 2.5	0.3 ± 0.6, − 0.2 to 0.7, *p* = 0.25
CL	1.3 ± 0.7	0.8 to 1.9	1.1 ± 0.9	0.5 to 1.8	0.2 ± 0.4, − 0.1 to 0.5, *p* = 0.18
OL	1.0 ± 0.9	0.4 to 1.7	1.1 ± 0.9	0.4 to 1.7	− 0.1 ± 0.6, − 0.5 to 0.3, *p* = 0.66
OM	0.8 ± 0.9	0.1 to 1.4	0.8 ± 0.8	0.2 to 1.4	0.0 ± 0.4, − 0.3 to 0.3, *p* > 0.99
Investigator 2					
SF	1.1 ± 0.6	0.5 to 1.2	0.9 ± 0.5	0.5 to 1.2	0.2 ± 0.4, − 0.1 to 0.5, *p* = 0.16
ST	1.0 ± 0.7	0.5 to 1.5	1.0 ± 0.8	0.4 to 1.6	0.0 ± 0.3, − 0.2 to 0.2, *p* = 0.83
CI	1.9 ± 1.0	1.2 to 2.6	2.1 ± 1.2	1.2 to 2.9	− 0.2 ± 0.5, − 0.5 to 0.2, *p* = 0.37
CL	1.3 ± 0.8	0.7 to 1.9	1.5 ± 0.8	0.9 to 2.0	− 0.2 ± 0.4, − 0.5 to 0.2, *p* = 0.28
OL	1.0 ± 0.8	0.5 to 1.6	1.2 ± 1.0	0.5 to 1.9	− 0.1 ± 0.6, − 0.5 to 0.3, *p* = 0.44
OM	0.9 ± 0.8	0.3 to 1.4	0.9 ± 0.7	0.3 to 1.4	0.0 ± 0.5, − 0.3 to 0.4, *p* = 0.90

### Main results

The means of the differences for the left and right sides of all the measured patients ranged from 0.8 to 1.3 mm, with an average of 1.1 ± 0.2 mm for all midfacial skeletal landmarks. The standard deviations ranged from 0.6 to 1.4 mm with a computed mean of 0.9 ± 0.3 mm (Table 4). There was no statistical difference in the means of differences between male and female patients.

**Table 4:** Means, standard deviations, and confidence intervals for the differences between both sides

Skeletal landmark	Mean ± SD (mm)	95% CI (mm)	Range of symmetry (mm)
Frontozygomatic suture	1.1 ± 0.9	0.9 to 1.3	0–2.0
Temporozygomatic suture	1.3 ± 1.4	1.0 to 1.5	0–2.7
Infraorbital canal	1.3 ± 0.9	1.1 to 1.5	0–2.2
Crista lacrimalis posterior	1.0 ± 0.8	0.8 to 1.1	0–1.8
Lateral orbita	0.8 ± 0.6	0.6 to 0.9	0–1.4
Medial orbita	0.9 ± 0.7	0.8 to 1.0	0–1.6

We compared the measured differences of every single CT scan to the computed values for a range of symmetry. All the skeletal landmarks were within the symmetry range in 41% of the examined individuals, showing perfect midfacial symmetry. For another 34% of examined individuals, there was conditional midfacial symmetry in that only one of the skeletal landmarks was not within the symmetry range. According to our definition of symmetry, this concludes that 75% of our population shows symmetrical proportions of the midface.

Another interesting finding was that the means for the measurements of the left and right sides showed higher values for the right side than the left side in all reference points but the most lateral point of the infraorbital canal. Even though the values do not differ much, there are statistically significant differences in measured means (*p* < 0.05) between the left and right sides in all reference points, except for the most lateral point of the infraorbital canal. There is an apparent deviation (tendency) in size towards the right side (Table 5).

**Table 5:** Means for the skeletal landmarks and their significance in differences of mean

Skeletal landmark	Mean ± SD right side (mm)	95% CI right side (mm)	Mean ± SD left side (mm)	95% CI left side (mm)	*p* value
Frontozygomatic suture	52.22 ± 2.58	51.71 to 52.73	51.51 ± 2.64	50.99 to 52.04	< 0.001
Temporozygomatic suture	59.92 ± 3.64	59.20 to 60.65	59.46 ± 3.41	58.79 to 60.13	< 0.001
Infraorbital canal	32.94 ± 2.35	32.48 to 33.41	33.17 ± 2.38	32.70 to 33.65	0.181
Crista lacrimalis posterior	17.08 ± 1.55	16.77 to 17.38	16.80 ± 1.81	16.44 to 17.16	0.01
Lateral orbita	47.53 ± 2.31	47.07 to 47.99	47.11 ± 2.35	46.64 to 47.57	< 0.001
Medial orbita	13.96 ± 1.72	13.62 to 14.30	13.53 ± 1.68	13.20 to 13.87	< 0.001

## Discussion

### Main findings

According to our definition, the cohort showed overall midfacial symmetry in 75% of individuals. Even though the measured midfacial skeletal landmarks showed, on average only minute differences between the left and right sides, the computed differences were significant except for the infraorbital canal.

Reproducibility testing showed no bias rendering the measuring method reliable. However, during testing for reproducibility, the infraorbital canal showed the highest differences between the left and right sides, suggesting an unfavourable variability for symmetry studies.

When measuring the proposed skeletal landmarks of the midface, symmetry can be assumed when there is a measured difference of 2.5 mm or less. The suggested methodology readily applies to any DICOM viewer and gives quick results when scanning for midfacial asymmetry.

### Strengths and limitations

First, the investigated population consisted of approximately equal parts of men and women, and the mean age of the examined patients was identical. Therefore, we conclude that a real-world population was drawn for measurement analysis.

Secondly, our study took two-dimensional measurements on CT scans to look for midfacial symmetry; thus, it can be considered conventional morphometry. A three-dimensional technique to look for differences in the whole surface or midfacial bones was not applied; thus, there was no consideration of geometrical morphometry. However, the two-dimensional measurements were drawn from a three-dimensional imaging technique, avoiding the usual limiting factors of two-dimensional studies, such as magnification, image distortion, and structural superimposition. A recent review suggests applying landmark-free three-dimensional quantitative geometric-morphometric methods to provide adequate data or models for treating facial asymmetry or surgical reconstructions [13]. Three-dimensional measurements or testing for spacious congruency was not performed in our study and constituted a limitation.

### Comparison with other studies

The population of our study was 101 individuals. Considering the literature that deals with facial bony symmetry, there are reports of the same size [14–17], of smaller [11, 18, 19], as well as larger study populations [20, 21]. Except for HINGSAMMER, all of the studies mentioned above were cross-sectional of character, which is also the case in our work.

There are almost equal parts of men and women in our study. A similar proportion was ascertained by other authors [15, 16, 21, 22]. Other works included a higher amount of either female [14, 20] or male [17, 19] patients. One study even reported males only [11], rendering a comparison to a general population obsolete. By assuring the exact distribution for gender, there is a validation of an accurate representation of a real-world population. Thus, our results are relatively transferrable to the general population of Europe, which boasted a median age of 43.7 years for Europe and 46.0 years for Germany in 2019 [23].

The range of ages of several works in the current literature best fits our presented study [15, 16, 22]. Interestingly, these studies also have a 50:50 proportion of male to female patients. Further reports set the cut-off for maximum age between 40 and 60 years [11, 14, 17, 19, 20], which omits the anatomic analysis for old and very old people.

The range of age in the aforementioned studies also affects the average age for the study populations. The report by JANSEN ascertained an average age of 57 ± 18 years [15]. An Italian research group reported mean ages of 45.3 ± 20.0 years for male and 50.8 ± 21.1 years for female patients [16]. Both reports match population sizes and show an even gender distribution, as discussed before. Another fitting cross-sectional study also addressing the symmetry of the zygomaticomaxillary complex found a mean age of 58 ± 19 years for both genders [22].

The quest to find an answer to the presence of symmetry or asymmetry has been a keen interest in science for a long time [7]. Facial symmetry is vital in terms of facial aesthetics and interpersonal communication. It is also the basis of many CAS procedures in modern operative medicine. There are surveys, usually not comprising more than 100 subjects under investigation, that deal with the superficial features of the face as well as bony structures and parts of the central nervous system [6, 22, 24–27]. Many research works have documented the alterations in both hard and soft tissues observed in patients following orthognathic surgery. However, the findings of these studies often fall short of adequately addressing soft tissue symmetry during bone reconstruction [28–30]. Even when the bony tissues have been reconstructed in prior surgeries, there may still be a need for augmentation due to lacking symmetry of facial soft tissues [31, 32]. Thus, even when the symmetry of the facial bones was reconstructed, one may not expect the soft tissues to be symmetrical after surgery.

In order to measure facial symmetry, there needs to be agreement on the correct measuring technique, thus paving the way to the definition of symmetry. According to DOBAI, defining a median plane using unpaired cephalometric points is more accurate than paired reference points. The N-ANS-PNS plane represents the ideal median plane [33] but can be substituted by other comparable planes in case of a lack of a reference point [34]. A systematic review reported higher reliability of landmarks on the median sagittal plane [35]. Due to practicality and ease, our presented work also used landmarks in the median sagittal plane.

For measuring bony midfacial symmetry, we define different skeletal landmarks. When performing the measurements, these landmarks are easy to point out in CT scans and include the frontozygomatic suture, the temporozygomatic suture, the infraorbital canal, and the posterior lacrimal crest. Of these points, the frontozygomatic suture, the infraorbital canal, and the posterior lacrimal crest have a range of symmetry of less than 2.2 mm, regardless of the distance between these points and the median plane. Therefore, these points are suitable for an easy-to-perform assessment of the symmetry of the bony midface because they are easy to find, and their ranges of symmetry are, on average, small.

There are reports of even lower values of less than one millimetre measuring the mean differences between bilateral linear measurements. However, the complete facial skeleton was considered, and the study is mainly aimed at looking into differences between different skeletal sagittal relationships [20]. Another report found a mean of differences between the left and right sides of the zygoma to be 1.6 mm measuring linear distances in three-dimensional imaging. This value is closer to our result and considers the exact anatomic location [17]. More works deal with the symmetry of the midfacial bony tissues. Unfortunately, these cannot be compared, as they compare anatomic structures employing segmentation or model construction and measuring for three-dimensional congruency [11, 16, 22].

One should draw particular attention to the crista lacrimalis posterior, which marks the transition zone that defines the boundary between the orbital floor and the medial orbital wall. This zone is paramount for producing well-matched PSI for orbital and midfacial reconstruction [36]. A study using three-dimensional models of patients verified good reliability for mirroring the healthy to the affected side for medial orbita and orbital floor fractures [15].

The most medial point of the medial orbital wall and the most lateral point of the lateral orbital wall, as landmarks for symmetry, are of high clinical relevance for reconstructive surgery of the orbita and the midface [37–39]. These points' symmetry range is low, making them highly relevant as checkpoints for midfacial bony symmetry. On the other hand, it is more difficult to define these points precisely in a CT scan because it is subjective to determine the most lateral and medial points of the orbita to a certain extent.

In our presented study, the statistical significances (*p* < 0.05) between the right and left side values at the frontozygomatic and temporozygomatic sutures, lateral and medial orbita, and posterior lacrimal crest indicate higher values of the right facial half in our study’s population. Various studies show statistically significant deviations in the size of the right and left side of anatomical structures for men and women [40, 41] and right-handed and left-handed people [42]. There may also be external factors, such as environmental stress, or internal factors, such as genetic stress leading to such a finding [6]. The tendency towards a larger right half of the face in the present work cannot be entirely explained due to the pseudonymisation of the CT scans and the scope of the study.

We defined the normal range for dynamic midfacial bone symmetry as a deviation between the left and right sides from 0 mm to the mean value plus one standard deviation for the selected skeletal landmarks. Subjects with a maximum of one reference point outside the defined normal range were considered symmetrical. Reports with a stricter definition of symmetry allow only differences of up to 2 mm and testify to a very high rate of symmetry in 94% of mirrored and unmirrored skeletal landmarks in subjects under investigation [43]. These findings are in accordance with the symmetry of 75% of our subjects under investigation using the mean difference plus one standard deviation. Another study suggested a maximum of 3 mm deviation for defining asymmetry in the zygomatic complex [11]. Again, this agrees with the 2.7 mm maximum difference between the left and right sides we ascertained.

We suggest a methodology to check facial bony symmetry by measuring the distances of the left and right sides to the median plane for at least three skeletal landmarks selected from the following: sutura frontozygomatica, sutura temporozygomatica, infraorbital canal, or posterior lacrimal crest. If all of the determined differences are equal to or lower than 2.5 mm, then the symmetry of the bony midface can be assumed, and symmetry-based methods for CAS procedures can be applied. If the values are higher than 2.5 mm for the mentioned skeletal landmarks, further planning and designing PSIs should proceed cautiously. At best, no extra correction is required, and traditional symmetry-based approaches may be used. If there is a substantial offset during the planning of the implant, manual corrections through manual adjustment or, for example, the use of a standardized skull [2] may have to be used.

## Conclusion

Static or strict symmetry does not exist in the human body. Average or standardised values of dynamic symmetry were challenging to define. Thus, we established an easy-to-perform methodology to prove the symmetry of a patient's bony midface before using symmetry-based CAS procedures. If the determined differences were equal to or less than 2.5 mm in the mentioned skeletal landmarks, then the symmetry of the bony midface was present, and symmetry-based methods for CAS procedures could have been applied.

Our results agree with the reports of other works and testify to the technique as a reliable instrument for checking symmetry. We also concluded that most patients in our study were suitable for symmetry-based CAS procedures.

## Data Availability

The datasets generated or analysed during the current study are available from the corresponding author upon reasonable request.
